# Mesoscale circulation determines broad spatio-temporal settlement patterns of lobster

**DOI:** 10.1371/journal.pone.0211722

**Published:** 2019-02-01

**Authors:** Paulina Cetina-Heredia, Moninya Roughan, Geoffrey Liggins, Melinda A. Coleman, Andrew Jeffs

**Affiliations:** 1 Regional and Coastal Oceanography Laboratory, School of Mathematics and Statistics, UNSW Australia, Sydney, Australia; 2 Department of Primary Industries, NSW Fisheries, Sydney, New South Wales, Australia; 3 Department of Primary Industries, NSW Fisheries and National Marine Science Centre, Coffs Harbour, New South Wales, Australia; 4 Institute of Marine Science, and School of Biological Sciences, University of Auckland, Auckland, New Zealand; MARE – Marine and Environmental Sciences Centre, PORTUGAL

## Abstract

The influence of physical oceanographic processes on the dispersal of larvae is critical for understanding the ecology of species and for anticipating settlement into fisheries to aid long-term sustainable harvest. This study examines the mechanisms by which ocean currents shape larval dispersal and supply to the continental shelf-break, and the extent to which circulation determines settlement patterns using *Sagmariasus verreauxi* (Eastern Rock Lobster, ERL) as a model species. Despite the large range of factors that can impact larval dispersal, we show that within a Western Boundary Current system, mesoscale circulation explains broad spatio-temporal patterns of observed settlement including inter-annual and decadal variability along 500 km of coastline. To discern links between ocean circulation and settlement, we correlate a unique 21- year dataset of observed lobster settlement (i.e., early juvenile & pueruli abundance), with simulated larval settlement. Simulations use outputs of an eddy-resolving, data-assimilated, hydrodynamic model, incorporating ERL spawning strategy and larval duration. The latitude where the East Australian Current (EAC) deflects east and separates from the continent determines the limit between regions of low and high ERL settlement. We found that years with a persistent EAC flow have low settlement while years when mesoscale eddies prevail have high settlement; in fact, mesoscale eddies facilitate the transport of larvae to the continental shelf-break from offshore. Proxies for settlement based on circulation features observed with satellites could therefore be useful in predicting broadscale patterns of settlement orders of magnitudes to guide harvest limits.

## Introduction

The influence of physical oceanographic processes on the dispersal of larvae is critical for understanding the ecology of species, evolution of marine communities, and for predicting settlement into fisheries to aid long-term sustainable harvest [1]. For most species with dispersive larval stages, however, we know little about the specific factors influencing dispersal during their time in the ocean. Despite an increase in studies using biophysical models to explore larval dispersal, the extent to which different factors such as ocean climate, or species larval traits, can explain patterns of settlement remains largely hypothetical. This is mostly due to the paucity of long term observed settlement data required to test the accuracy of biophysical models that seek to represent settlement. Comparison of biophysical model estimates against long-term observations can be a powerful tool for discerning the key factors involved in structuring patterns of settlement which often drives subsequent recruitment to important fisheries [2]. Indeed, the ability to predict the arrival of larvae onshore and subsequent recruitment into a fishery can aid in setting and adapting harvest limits effective for sustainable management [3–5].

Palinurid lobsters, commonly known as spiny or rock lobsters, are key components of coastal ecosystems [6, 7], and form the basis of highly lucrative fisheries in many parts of the world with a combined global value of harvests in excess of US$1 billion a year [8]. As a result of their high market value, dedicated fisheries, often with intense harvesting pressure and with specific management focus, are common features for most spiny lobster species [9]. Spiny lobster populations worldwide display dramatic inter-annual and spatial variability in settlement and recruitment [3, 10]. The relationship between settlement abundance and stock populations for some lobster species, such as *Panulirus argus*, distributed across extensive coastlines of the Atlantic, is hindered by post-settlement processes [11–13]. However, for other species, direct observations of recruitment to the fishable stock have been related to settlement of post-larvae (pueruli), suggesting that settlement provides a leading indicator of the abundance of lobsters several years later [14–17]. For instance, in Western Australia, pueruli settlement of *Panulirus cygnus*, western rock lobster, is correlated with adult abundance 4 years later, and annual measures of pueruli settlement are used as a fundamental variable in managing subsequent landings [14]. The strength of pueruli settlement has therefore utility in predicting recruitment and calculating harvest limits [15]; consequently, long-term monitoring of lobster settlement is commonplace [14, 18, 19]. An understanding of drivers of pueruli settlement will aid improved prediction of settlement and its variability over large spatial scales, thereby guiding future monitoring programs and management of lobster fisheries.

*Sagmariasus verreauxi* (Eastern Rock Lobster, ERL) inhabit a stretch of uninterrupted coastline along southeast Australia. In this region, the East Australian Current (EAC), a swift Western Boundary Current (WBC), meanders poleward aligned with the coast year-round, feeding eddies and driving the regional main patterns of circulation [20]. The EAC separates from the continent typically between 30.7–32.4°S but can extend further south to 38°S [21]; the formation and shedding of mesoscale eddies throughout the year displace the EAC separation latitude [21, 22], and influence the regional circulation with a ~120 days periodicity [23, 24]. A previous modelling study of ERL dispersal under contemporary and future climate conditions in the EAC proposed that a future poleward displacement of the mean EAC separation latitude will induce a similar poleward shift of maximum settlement [25]. However, a quantitative validation against observations of the extent to which the EAC determines settlement and its variability along southeast Australia remains unknown.

Here, we quantitatively test whether observed settlement of ERL along southeastern Australia can be estimated from larval dispersal simulations that rely on mesoscale circulation and pelagic larval duration. ERL settlement has been measured for 21 years along this coast, providing an invaluable data set for validation of simulated settlement and for discerning the relative importance of physical processes on pueruli settlement. We use outputs of an eddy-resolving hydrodynamic model with data assimilation to simulate larval dispersal over a 21 year period. The larval trajectories are used to: 1) examine the role of ocean currents in explaining observed spatial and temporal variability of ERL settlement, and 2) diagnose circulation features facilitating larval supply to the continental shelf-break in this dynamic region.

## Methods

### Study species

*Sagmariasus verreauxi* (Eastern Rock Lobster, ERL), has a life cycle with benthic adults living in coastal waters and a pelagic larval phase spent in open ocean. Hatching of eggs, carried by adult female ERL occurs simultaneously across spawning grounds (i.e., in southeast Australia between 28°-33°S), from November to January peaking in December (G. Liggins pers. comm.). The hatched naupliosoma larvae moult into phyllosoma larvae and drift in the ocean for 8–12 months before metamorphosing into pueruli that can actively migrate across the shelf seeking suitable habitat in which to settle [26, 27]. The subsequent recruitment of juveniles re-stocks the adult population, which shows no genetic differentiation along southeast Australia [28].

### ERL puerulus monitoring program

**Fig 1:**
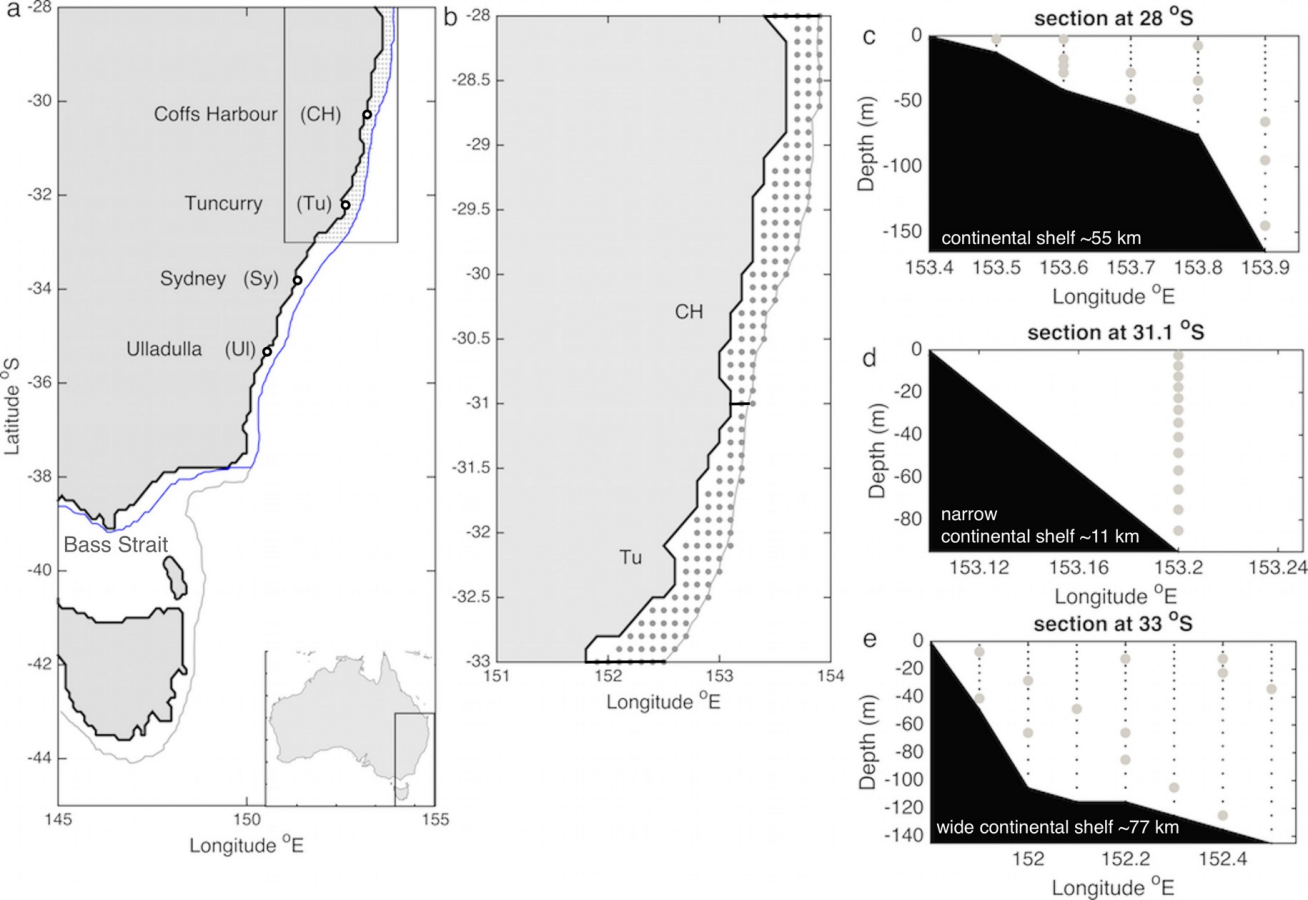
a) Map of the study area showing the settlement monitoring locations Coffs Harbour (CH), Tuncurry (Tu), Sydney (Sy) and Ulladulla (Ul) indicated by the empty circles; the 150 m isobath is shown in grey and the area within which settlement was consider to occur is delimited by the blue line (i.e. 150 m isobath along the southeast coast of Australia and the 50 m isobath along the south coast of Australia). b) Top view map encompassing spawning grounds; the grey dots show a top view of all possible release locations and sections shown in panels c-e are indicated with black thick lines. c-e) vertical sections across 28, 31.1 and 33°S, where the continental shelf is ~55 km, the narrowest (~11 km) and the widest (77 km) respectively, showing the depth distribution of release locations (grey dots) for a day (1 Dec 2010) as an example. Black dots indicate all possible release locations.

In 1995 the Department of Primary Industry Fisheries of New South Wales (NSW, 28–37°S) established an annual survey to monitor the abundance of ERL pueruli recruiting to the coast. One of the general objectives was to understand the spatial and temporal variability in recruitment and the influence of environmental factors on settlement [26], [29]. As part of the monitoring program ERL pueruli collectors have been deployed to survey settlement at four locations (three replicate sites within each location with three collectors at each site) extending along the coast of south-east Australia: Coffs Harbour (30.3°S), Tuncurry (32.1°S), Sydney (33.8°S), and Ulladulla (35.4°S) [26], (Fig 1).

The collectors and their use in the long term pueruli settlement surveys are thoroughly described in [30]; briefly, they are sea-weed-type collectors sampled every 4 weeks during the first quarter of the lunar month from August to January. Monthly settlement at each location is estimated using the mean number of pueruli captured in the collectors.

The ERL monitoring program has produced a 21-year time series of ERL settlement along a latitudinal range extending over 500 km. The number of pueruli settling at each collector has been recorded and used to compute a mean number of pueruli settled at each location and survey month (from August to January within each year). This study examines inter-annual variability in settlement along ~500 km of coastline along southeast Australia using yearly settlement. Yearly settlement is derived from the summation of monthly settlement measured from all pueruli collectors for each of the four locations from August to January. For example, pueruli settlement surveyed between August 1995 and January 1996 is considered to contribute to settlement in 1996.

### Hydrodynamic model

ERL larval transport by ocean currents is simulated by advecting larvae with velocity outputs from the Ocean Forecasting Australian Model Bluelink ReANalysis (OFAM BRAN 3p5). This is a near-global hydrodynamic model with data assimilation developed to hindcast and forecast realistic upper ocean conditions. BRAN3p5 has a spatial horizontal resolution of 0.1° for all longitudes between 75°S and 75°N that allows it to resolve eddies, and 51 z* vertical levels with a 5 m vertical resolution in the first 40 m of water depth, 10 m vertical resolution to 200 m deep, and a decreasing vertical resolution spanning 120–1000 m between vertical layers from 200 m to the sea floor [31]. To better represent bottom topography a ‘partial cell’ scheme (z*) is implemented [32].

OFAM BRAN 3p5 assimilates sea surface temperature (SST), sea level anomaly (SLA) and in-situ temperature and salinity observations to regularly adjust the model state to match the observations. Data assimilation is particularly useful in eddy-resolving models because the generation and evolution of eddies is modulated by unpredictable instabilities; therefore data assimilation is useful in order to reproduce eddies in the correct place and time or with accurate intensity and characteristics [31]. The design of BRAN 3p5 allows it to reliably reproduce mesoscale eddies which are known to influence larval dispersal [25, 33], and therefore this model is an ideal tool to study larval dispersal.

### ERL larval transport simulations

Larval transport simulations are conducted with the Connectivity Modelling System, CMS, [34], a Lagrangian tracking model. Because the study focuses on the impact of ocean circulation on larval transport and consequent settlement, the Lagrangian simulations represent passive larvae and do not account for spatio-temporal changes in breeding stock abundance and productivity. Nevertheless, particle release, particle advection time, and rules for settlement are based on the ERL habitat, life cycle, and larval phase duration. Specifically, particles are seeded along the natural spawning grounds from 28 to 33°S and between the coastline and the 150 m isobath daily from 15 November until the 15 January; data supplied by NSW commercial lobster fishers indicate that, during the period 2009–2017, >97% of the reported catch of ovigerous females occurred within these latitudes and depths; and data from a NSW fishery-independent survey indicates that 90% of ovigerous females shed their eggs during December (G. Liggins pers. comm.). Larvae are advected with simulated ocean currents throughout their pelagic stage (8–12 months, [26] and all larval trajectories are recorded over 12 months. As a proxy for settlement we model the delivery of larvae onto the continental shelf-break due to the narrow shelf width (as low as 16 km within the study domain) and the model spatial resolution (0.1°). After hatching, ERL larvae take 8–12 months to develop into a pueruli competent to settle. Thus, larvae are considered to settle if at any time 8–12 months after release they are anywhere between the coastline and the 150 m isobath along the east coast, or between the coastline and the 50 m isobath within Bass Strait (Fig 1A); the trajectory of larvae after settlement is ignored. Due to paucity of data on larval survival in the ocean for larvae of any lobster species [35], we assume mortality is spatially and temporally uniform.

The vertical distribution of lobster larvae in the ocean has been examined for some species; the western lobster, *Panulirus cygnus*, migrates vertically to increasing depths with age [36]; similar changes in the vertical distribution of the southern rock lobster, *Jasus edwardsii*, have been observed with mid-stage larvae concentrated within the top 20 m and late stage larvae moving down to 100 m [37]. It is expected that larvae of this study species, *Sagmariasus verreauxi*, undergo vertical migration; however, due to lack of empirical data describing their vertical distribution, the larval transport simulations do not implement specific vertical migration. Instead, they allow for passive vertical movement using 3D velocity fields for advection as well as an initial distribution encompassing all depths. Dispersal studies have shown larval behavior can impact settlement [38]; for instance, [39] finds that ontogenetic vertical migration of the Caribbean lobster, *Panulirus argus*, larvae diminishes dispersal distances and increases settlement abundance. This is partly due to differences in current direction or speed with depth [40]. However, unlike the ocean region inhabited by *P*. *argus* larvae, the dominant circulation features in the study region, such as the EAC and mesoscale eddies, are all coherent with depth; with the EAC prevailing over thousands of meters [41], and mesoscale eddies extending over 100s of meters from the surface which is the primary habitat of these larvae [42, 43]. Thus, main circulation features are expected to induce similar particle trajectories irrespective of their depth distribution; particularly, over the top 200 m of the water column, a range within which the larvae of all rock lobster species have been found to reside regardless of developmental stage ([35]. In the region, [44] argue that surface dispersal patterns of king prawn along southeast Australia would not change significantly from those at depth. By simulating the dispersal of larvae without behavior we test the extent to which advection by ocean currents can explain observed broad patterns of settlement. Implementation of larval behavior however, could improve settlement predictions from simulations if empirical data were available to inform models.

We released the same number of particles at each source-latitude (i.e., every 0.1° between 28–33°S) and at the centre of the grid cells of the hydrodynamic model; the number of particles seeded at each source-latitude is determined by the number of grid cells between the coastline and the 150 m isobath at the latitude where the continental platform is the narrowest (i.e., 13 grid cells at 31.1°S). The release longitudes and depths at each 0.1° of latitude are randomly chosen daily amongst all possibilities (i.e., throughout the water column and at any longitude between the coastline and the 150 m isobath). Because field observations of the vertical distribution of larvae are lacking, we released particles at random depths but setting a condition that warranted particles being released throughout the water column rather than mostly at shallow depths; i.e. particles releases must occur below and above 50m. Across any given latitudinal section from the coast to the 150 m isobath, there are more possible release locations at shallow than at deeper waters. Thus, we chose a 50 m threshold depth to ensure releases occurred at most depths throughout the water column (Fig 1C–1E). We seeded 13 particles at each 0.1° of latitude from 28–33°S; this implies that 663 particles were released daily for a total of 40,443 particles over a spawning season. Larval transport predictions can be sensitive to the number of particles released (e.g. [45] [46]; a previous study of dispersal in the region corroborates that settlement patterns obtained from dispersal simulations of ERL larvae do not change beyond 455 virtual larvae released per day [25]. The number of releases in the present study is above such threshold. Particles are advected offline with a time-step of 1 hour and with the 3D velocity fields generated by the hydrodynamic model plus a random velocity vector to account for circulation structure at spatial scales smaller than that captured by the hydrodynamic model (i.e., sub-grid scale diffusion). The velocity vector associated with diffusion is scaled by a horizontal diffusion coefficient set to 8.8 m^2^ s^-1^ following [47] which has been previously used in dispersal studies with similar hydrodynamic model outputs [25].

### Spatial and temporal variability analysis

#### Comparison of observed and simulated settlement

Total simulated larval settlement along the coast of southeastern Australia was obtained for each year between 1995–2015 and each 0.1° of latitude; settlement at each 0.1° of latitude was measured as the total amount of larvae that reached any grid cell between the coast and the 150 m isobath at any time 8–12 months after release. Although monthly variability in settlement has been observed, this study focuses on a larger temporal scale, i.e., annual and longer.

The ratio of simulated settlement north and south of 32°S is compared against the ratio of observed settlement in northern (Coffs Harbour and Tuncurry) and southern (Sydney and Ulladulla) locations. North and south simulated settlement is obtained adding settlement occurring at all grid cells north and south of 32°S. Observed north settlement is obtained adding monitored settlement in Coffs Harbour and Tuncurry and observed south settlement is obtained adding monitored settlement in Sydney and Ulladula.

In addition, inter-annual variability of observed settlement summed across all monitored locations is compared against the addition of simulated settlement occurring within all four 0.1° latitude degree closest to the monitored locations (i.e., Coffs Harbour 30.3°S, Tuncurry 32.3°S, Sydney 33.9°S, and Ulladulla 35.5°S). Observed and simulated annual settlement are also compared separately at individual locations in order to discern the spatial scale over which settlement estimates agree with observations (e.g., along southeast Australia or at specific locations). To correlate observed and simulated inter-annual settlement, yearly settlement is first normalized by total settlement across years of observations and simulations respectively. That is, yearly settlement is divided by total settlement over the 21 years and multiplied by 100. Non-oceanographic processes such as a gradual change in size of the breeding stock over time can cause long-term variation in observed settlement. To minimise the effect of such long-term factors on our analysis, we compute yearly changes in settlement and correlate the observed and simulated time series of year-to-year differences in settlement. If inter-annual differences in settlement due to ocean currents are much greater in magnitude than inter-annual differences, due to changes in the abundance of breeding stock and egg production then this approach effectively removes the confounding influence of long-term changes in egg production from the analysis. This approach therefore examines the importance of dispersal by ocean currents in determining year-to-year variations in settlement. Changes in yearly settlement are computed from the normalized yearly settlement time series of observed and simulated settlement respectively.

To examine dominant periodicities or temporal variability over the 21-years, a wavelet analysis is conducted on both the observed and simulated time series of yearly normalized settlement. The wavelet analysis is computed using code from [48], and utilising the bias correction of [49]. Finally, to elucidate if there is a single or more ERL population(s), a mean connectivity matrix was composed across the 21 years; each year, the number of larvae released at each source latitude and arriving to each sink latitude was quantified. Source latitudes span 28–33° S every 0.1° and sink latitudes 26–38° S every 0.1°.

#### Modelled ocean circulation and simulated settlement

To interpret settlement in the context of ocean processes, the spatial variability of yearly settlement along the coast obtained from simulations is compared to the variability of the modelled circulation along the coast. To account for circulation variability we focus on two factors; 1) the proximity to the shelf break of a coherent unidirectional flow (i.e., EAC), and 2) the spatial structure of persistent flow identified with empirical orthogonal functions (EOFs).

Here, we explore if regions where the EAC is far from the coast, separates from the coast, or has a dominant component perpendicular to the coastline, coincide with regions of simulated and observed higher settlement. With this purpose we detect daily locations where the EAC departs from an alongshore path as it travels polewards over a time period encompassing the direct observations from the long-term pueruli survey program (1995–2015, Fig 2A).

**Fig 2:**
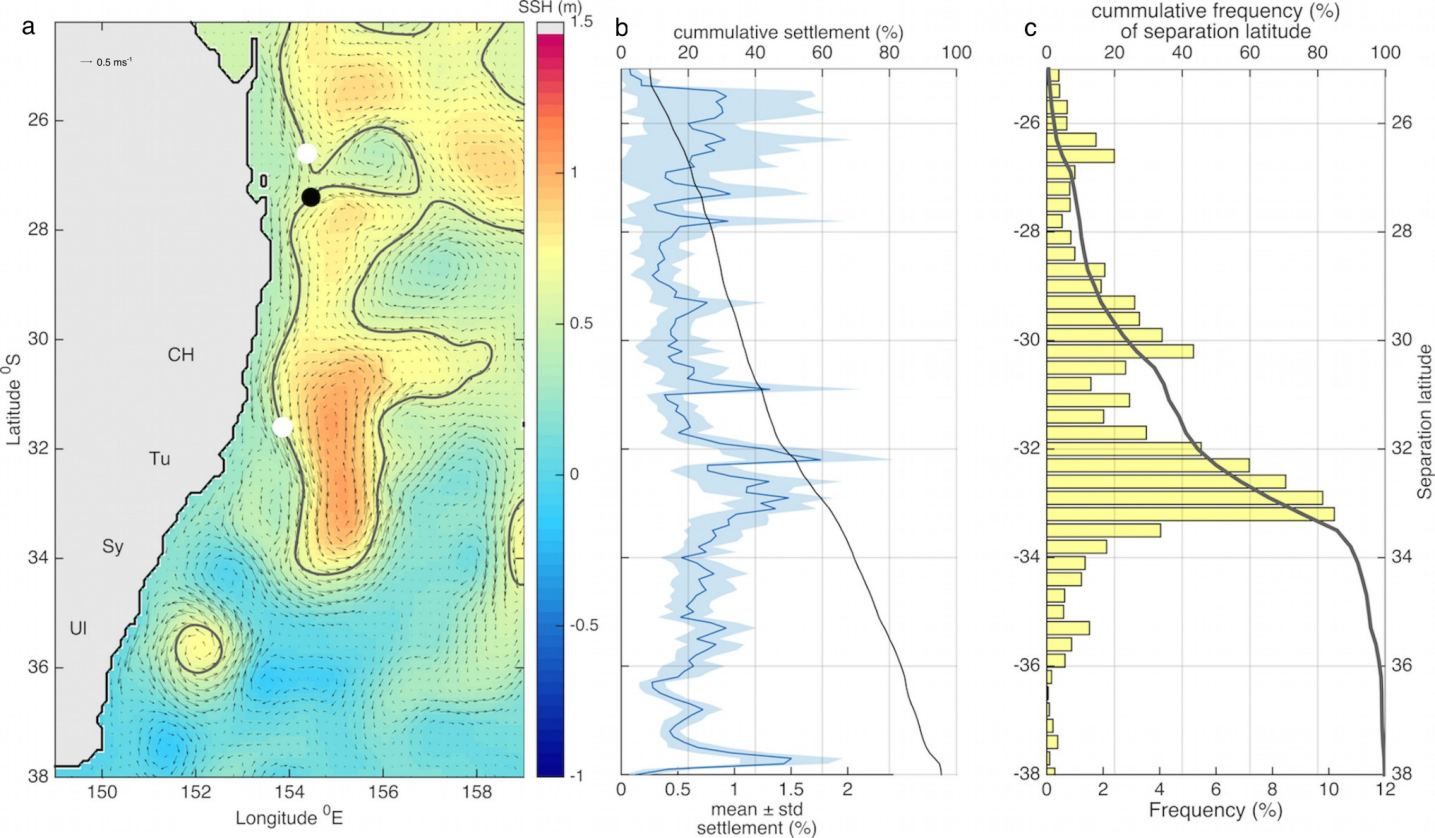
a) Map depicting the EAC meandering as it flows polewards and separating from the continent. Snapshot of modelled Sea Surface Height (SSH, colorbar), and the EAC SSH isoline (black contour); the white dots indicate latitudes where the EAC deviates from the 150 m isobath for more than 70 km; the black dot indicates where the EAC reattaches (comes within 70 km from the 150 m isobath after having deviated upstream), the arrows represent the geostrophic currents derived from modelled SSH. b) Across years mean (thick line) and standard deviation (blue shade) of the percentage of settlement at each 0.1°of latitude relative to total settlement across latitudes. The black line shows cumulative settlement from north to south. c) Frequency histogram of the EAC separation latitude over 21 years (1995–2015, bars) and cumulative frequency from north to south (black line).

Specifically, we record the latitudes where the core of the current is more than 70 km eastward of the 150 m isobath or farther, as well as locations where the stream re-attaches or comes within 70 km of the 150 m isobath (Fig 2A). To identify the EAC core we find the Sea Surface Height (SSH) isoline corresponding to the maximum poleward geostrophic velocity at 25°S between the coast and 160°E. This approach is based on the method used [21] to identify the core of the jet and the latitude where the EAC separates from the continent.

A qualitative comparison between circulation structure and annual settlement is used to discern flow patterns associated with settlement for individual years; particularly, to recognize if there is a common flow pattern for years that show either high or low settlement. The Empirical Orthogonal Functions (EOF’s) capture the dominant modes of variability of the circulation; for instance, the prevalence over time of the EAC, or an eddy field. EOF’s are therefore used to identify the dominant circulation structure of each individual year. To compute the EOF’s we use surface velocity fields over the settling time period of each year; this analysis is designed to reveal the dominant circulation mode during the delivery of larvae to the continental shelf-break rather than that dictating trajectories throughout the pelagic stage, or across the 21-years of observed settlement. For instance, settlement in 1996, which corresponds to larvae hatched in November 1994-January 1995, is qualitatively compared against EOF’s calculated from velocity fields 8–12 months after being hatched (from July 1995 to January 1996). Finally, to further test the role of eddies as a mechanism facilitating settlement, we computed the proportion of larvae transported into the continental shelf-break by an eddy relative to total settlement for a specific year (2003).

## Results

### Spatial variability

#### Latitudinal variability in observed and simulated settlement

Observed settlement over the last 21 years (1995–2015) consistently shows low settlement in northern locations (Coffs Harbour at 30.3°S and Tuncurry at 32.3°S) and high settlement in southern locations (Sydney at 33.9°S, and Ulladulla at 35.5°S); the mean across years of the south to north observed settlement ratio is 11.9°8.4 S.E. Simulated settlement also reveals higher settlement in south compared to north locations every year; nevertheless the south to north ratio, based on simulated settlement north and south of 32°S, (1.85±0.83 S.E.) is almost an order of magnitude smaller than the observed.

**Fig 3:**
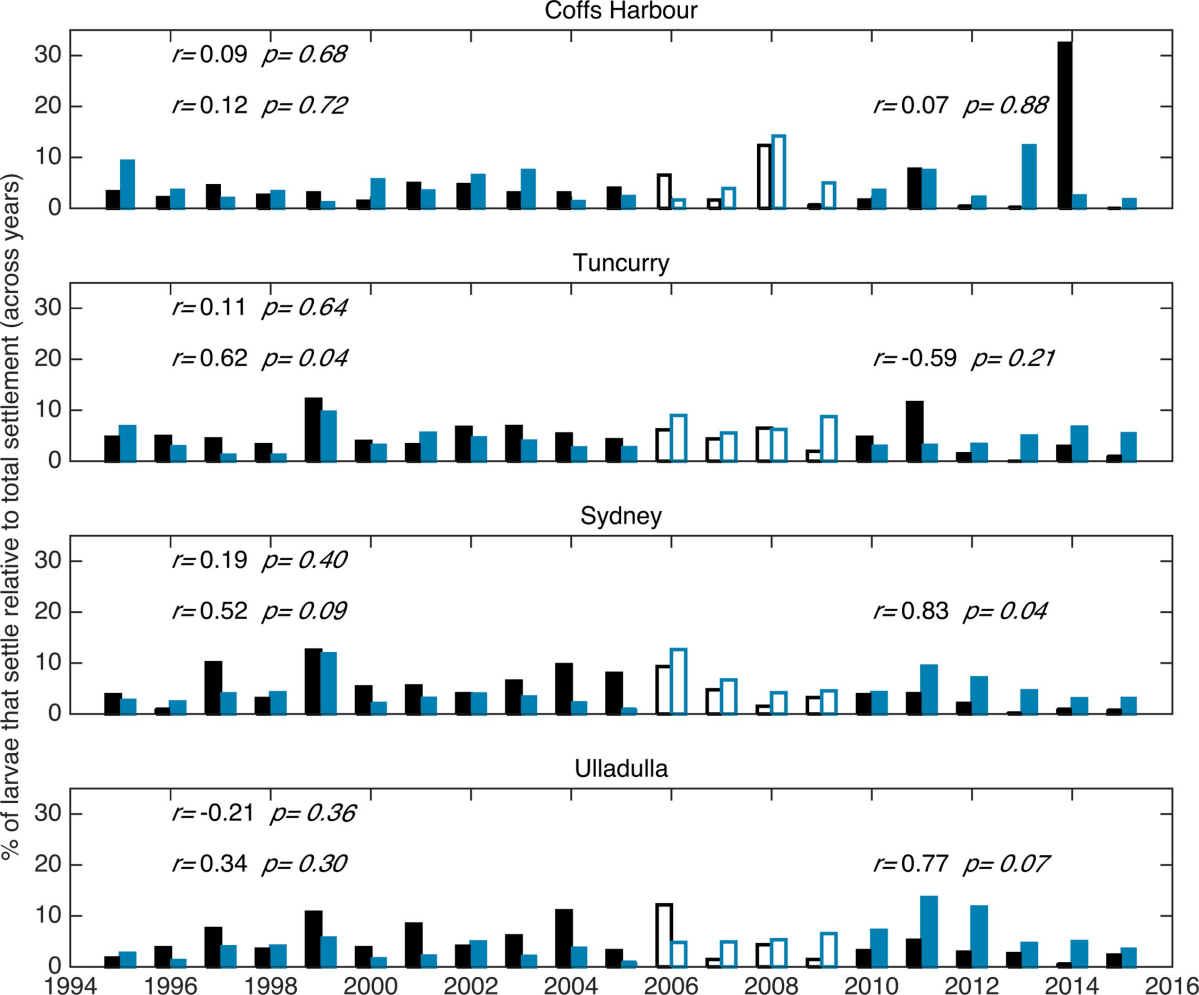
Twenty-one year (1995–2015) time series of observed (black) and simulated (blue) settlement at each monitoring location, correlation coefficients (r) and corresponding *p* values are shown for correlations between 1995–2015 (top left of each panel), 1995–2005 (mid-left), and 2010–2015 (mid-right). Open bars are used for 2006–2009 to facilitate distinction of decadal periods before and after, for which correlation coefficients and *p* values are included.

Despite evident inter-annual variability, there is a distinct pattern of low simulated settlement (<20% relative to total settlement) between 28–32.3°S (encompassing the northern locations where settlement is surveyed), high settlement (>20% relative to total settlement) between 32.4–36°S, (encompassing the southern locations where settlement is surveyed) and maximum settlement between 32.4–33.8°S (>30% relative to total settlement, Fig 2B). This broad pattern is in agreement with observed settlement patterns. At individual monitoring locations however, 21-year long time series of observed and simulated settlement are not significantly correlated (Fig 3).

#### Latitudinal variability in simulated settlement, EAC meandering and separation

The latitudinal range of simulated maximum settlement lies within a region where the core of the EAC often deviates from an alongshore path and is not in close proximity of the coast. In fact, a cumulative frequency histogram shows that within the region where the simulated settlement is highest (~32.4–33.8°S), the EAC core is at least 70 km east of the 150 m isobath 40–88% of the time over 21 years (1995–2015, Fig 2C); thus, maximum simulated settlement occurs within typical EAC separation latitudes.

### Temporal variability in observed and simulated settlement

**Fig 4:**
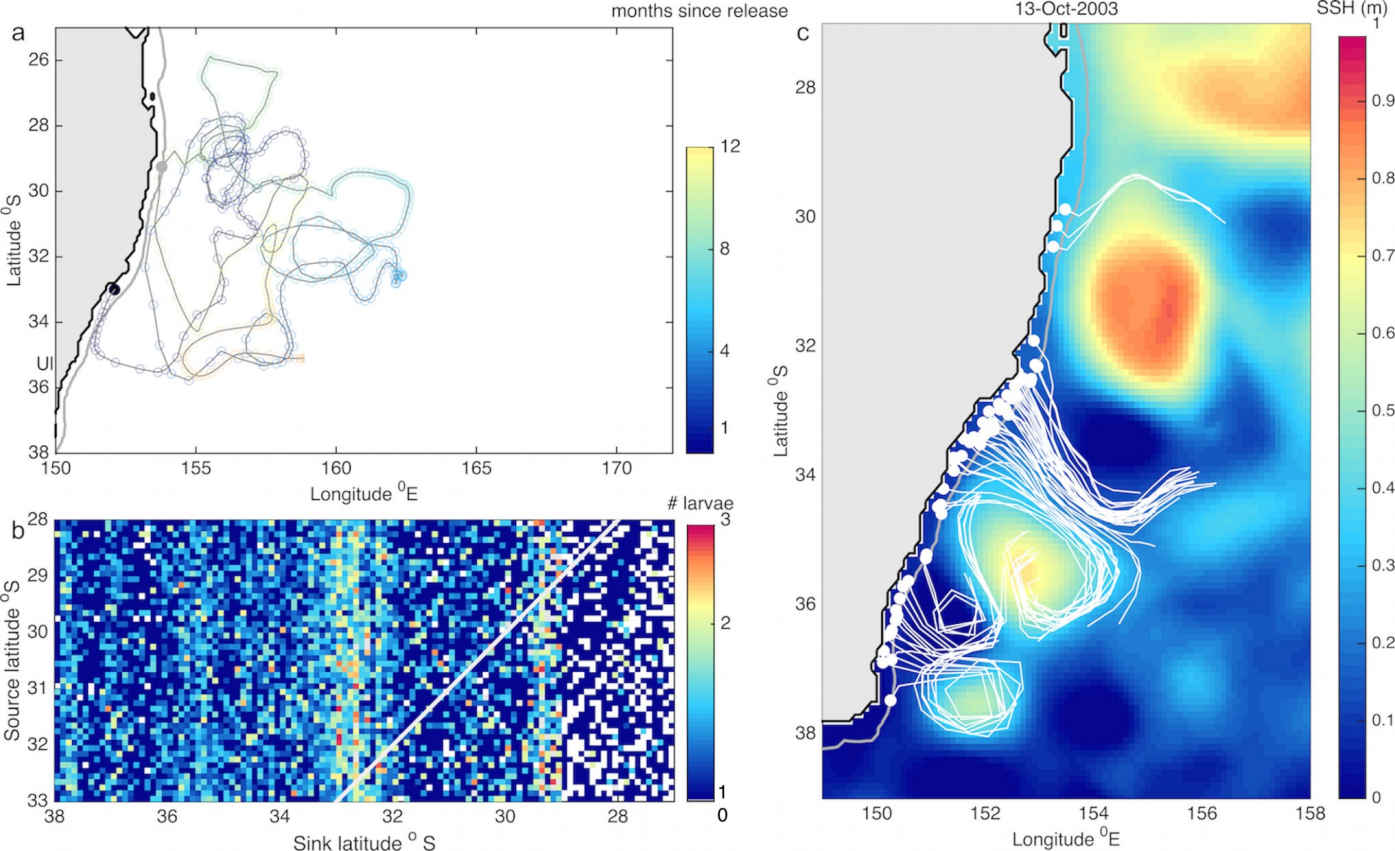
a) Twenty-one year (1995–2015) time series of observed (black) and simulated (blue) settlement added across monitoring locations, correlation coefficients (r) and corresponding *p* values are shown for correlations between 1995–2015 (r = 0.1, *p>>0*.*05*), 1995–2005 (r = 0.78, *p < 0*.*01* filled bars), and 2010–2015 (r = 0.78, *p = 0*.*065* filled bars). Open bars are used for 2006–2009 to facilitate distinction of decadal periods before and after, for which correlation coefficients and *p* values are included. b) Time series of observed and simulated yearly change in settlement, correlation coefficient (r = 0.63, *p < 0*.*01*). c) Observed vs. simulated yearly differences in settlement; filled symbols indicate years when observed and simulated differences agree (i.e., either both increase or decrease), while empty symbols indicate when they show opposite trends (i.e. one decrease and the other increases and vice versa); years with largest discrepancy are emphasized with an asterisk. Settlement and differences in settlement are presented in percentage relative to total settlement across all years for the observed and simulated settlement respectively.

Over the 21 years (1995–2015), observed and simulated settlement summed across all four locations (i.e. Tuncurry, Coffs Harbour, Sydney, Ulladula) and corresponding latitudes in the model respectively fluctuates over an order of magnitude; specifically, between 1.9–10.9% and 0.7–12.5% of the total settlement across the 21 years for the observations and simulations respectively (Fig 4A). Thus, the degree of variability along southeast Australia in observed settlement is well matched by the simulations.

The correlation between observed and simulated annual settlement (i.e., total settlement each year) over the entire 21 years is not significant at individual monitoring locations or latitudes (Fig 3) or for all for monitoring locations or latitudes summed together, with the simulation only explaining 1% of the variation in annual settlement for data summed across all four monitoring locations or latitudes (Pearson’s test, r = 0.11, *p = 0*.*61*, Fig 4A). It is noteworthy that the time series of observed and simulated settlement before and after 2007–2008 oscillate together; in fact, correlations between observed and simulated annual settlement over these two time periods (1995–2005, and 2010–2015) are high, explaining 61% of the variation (both with r = 0.78, *p < 0*.*01* and *p = 0*.*07* respectively, Fig 4A). In contrast, year-to-year changes in simulated settlement (20 data points), a measure of annual settlement partially corrected for long-term changes in breeding stock size, explain 40% of the year-to-year observed variation (i.e., increase or decrease in settlement from one year to the next), and are significantly correlated (r = 0.63, *p < 0*.*01*, Fig 4B).

**Fig 5:**
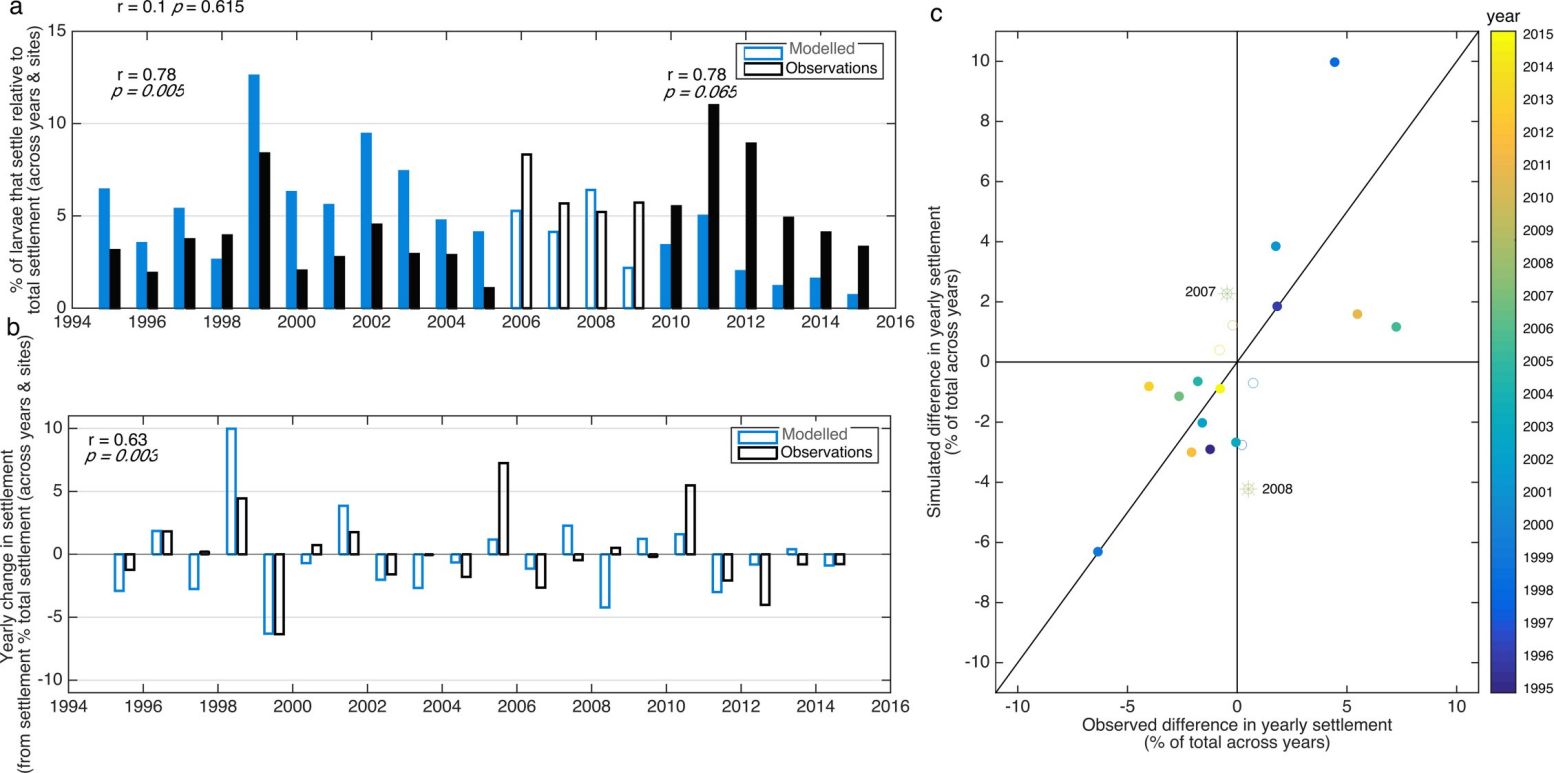
Global wavelet spectrum of simulated and observed settlement showing peaks of energy between 2–3 years and at 4 years.

Importantly, years of observed and simulated highest and lowest settlement coincide. For instance, 1999 and 2011 are years characterised by high settlement while 2005 and 2015 are characterized by low settlement, and this is well captured in the simulations (Fig 4). Additionally, a wavelet analysis identifies peaks of settlement occurring every four years in both the observed and simulated 21-year time series of total settlement (Fig 5).

## Discussion

This study uses an ocean hydrodynamic model coupled with a particle tracking model to simulate the dispersal of ERL larvae. Comparison against 21 years of larval settlement observations allows diagnosis of physical mechanisms driving larval dispersal and supply to the shelf-break, and quantifying the extent to which hydrodynamic processes determine patterns of settlement variability at different temporal and spatial scales. Despite the large range of factors that can impact larval dispersal, we show that along southeast Australia, within a Western Boundary Current (WBC) system, mesoscale circulation explains broad (inter-annual to decadal) spatio-temporal patterns of observed settlement of our model species *Sagmariasus verreauxi* (Eastern Rock Lobster, ERL) over 500 km of coastline.

**Fig 6:**
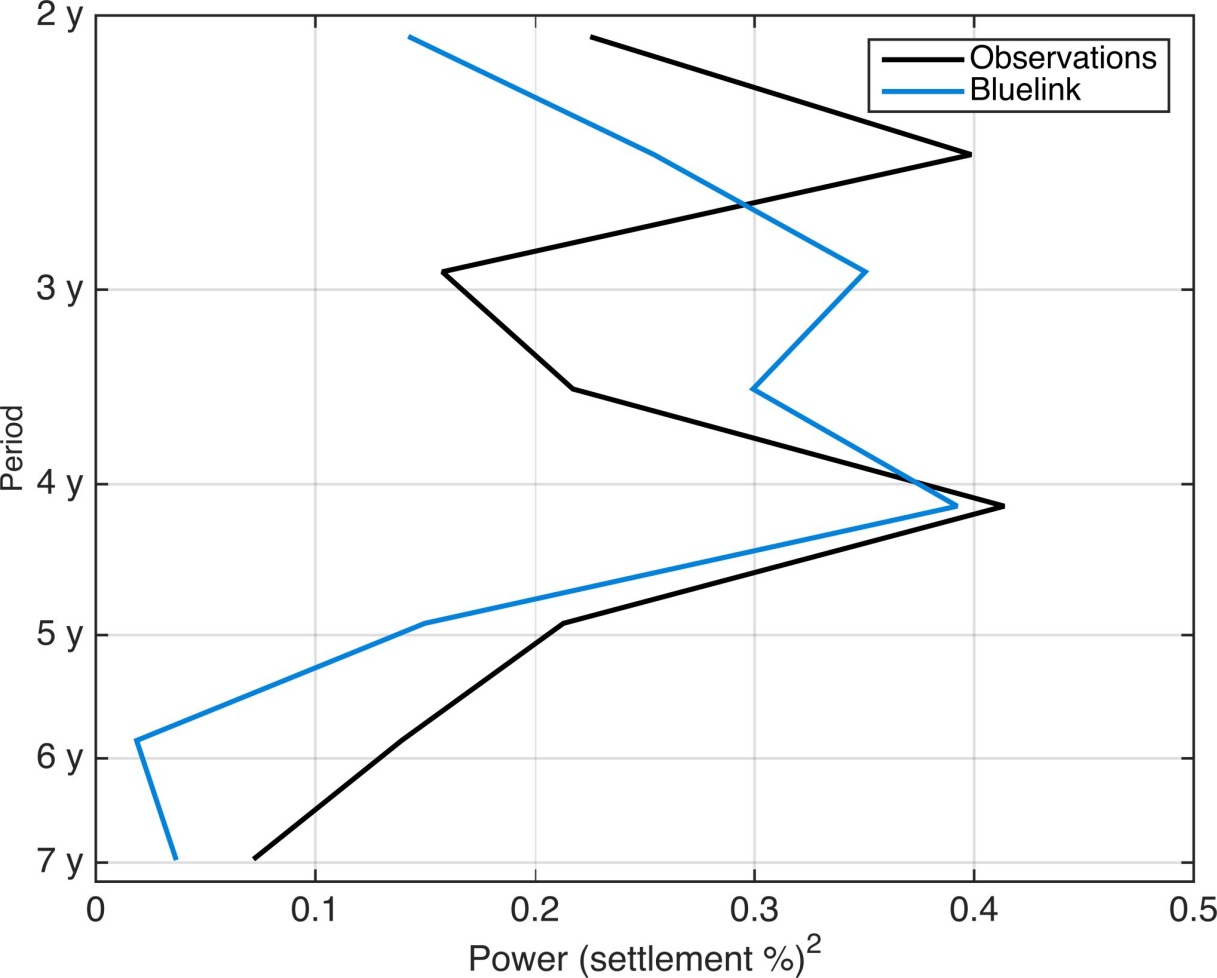
a-b) Spatial structure of the first mode of the Empirical Orthogonal Functions (EOFs) showing speed (colorbar) and velocity fields (arrows) for settlement time period in 1999 (a) and 2005 (b). Superimposed is settlement at each 0.1 latitude degree (grey line). c-d) temporal structure of the first mode of the Empirical Orthogonal Functions (EOFs) for the settlement time period in 1999 (c) and 2005 (d); the left hand axis shows settlement over time across all latitudes (grey dots).

Previous studies have proposed a number of mechanisms driving larval dispersal in the region [25, 33]; using observations of lobster settlement over a 21 year period, this study corroborates for the first time the impact of such mechanisms on larval delivery to the coast by comparing simulated with observed settlement. In agreement with the hypothesis proposed by [25], we demonstrate that patterns of settlement abundance are influenced by the presence and coherence of the poleward flowing EAC and the region where it deviates from the coast. More specifically, low and high settlement locations coincide with regions upstream and downstream of the latitude where the EAC separates (Fig 2); similarly, years with low settlement occur when a coherent EAC persists, while high settlement relates to a flow dominated by eddies, or flow perpendicular to the shelf break caused by a discontinuous EAC, EAC meandering, or separation from the continent (Fig 6). In addition, meso-scale eddies which often form where the EAC veers east, but are also present intermittently all along the coast as far north as 25°S, are a mechanism facilitating the transport of larvae onshore into the continental shelf-break.

Nevertheless, simulations based solely on the advection of passive larvae by mesoscale circulation do not capture observed settlement at smaller spatial scales (i.e., individual monitoring locations). Intricacies including exact differences in settlement abundance at individual monitoring locations might only be resolved when implementing the impact of local forcing, sub-mesoscale scale circulation, swimming behaviour of pueruli, and the interaction and effects of the environment on larval survival. This is further discussed in the model limitations section below.

### Spatial variability in settlement

#### Effect of the EAC jet, meandering, and separation on settlement

**Fig 7:**
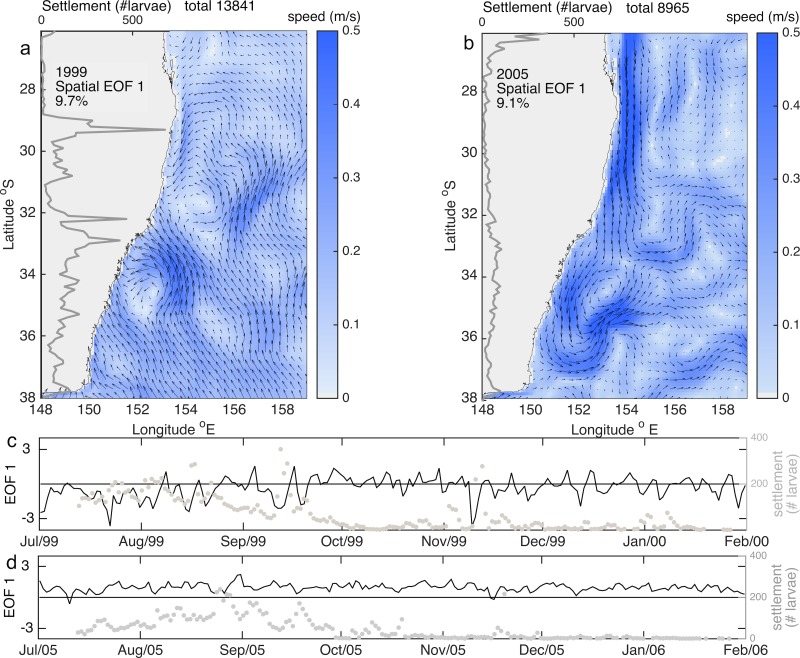
a) Trajectory of a particle that settles upstream, the black and grey dots show the release and settlement locations respectively, the particle trajectory is shown for 12 months rather than being truncated at settlement, i.e., at ~9 months after release). b) Mean connectivity matrix (1995–2015), the white indicates values that correspond to self-recruitment (the source and sink latitudes are the same), the colorbar indicates the number of larvae in logarithmic scale. c) Particle re-circulation within eddies and transport into the continental shelf break. Snapshot on the 13/October/2003 showing a simulated settlement pulse and preceding particle re-circulation within eddies with Sea Surface Height (SSH) on the background, dots indicate the position of particles at settlement and lines show their trajectories in the previous 10 days; only particles that reach settlement are shown.

The EAC facilitates advection of larvae along the coast and connectivity amongst locations separated over large distances, while its meandering and pinching off of eddies induces larval re-circulation and self-seeding. Although connectivity from upstream into downstream locations is dominant, there is also some larval exchange occurring from south to north (Fig 7A and 7B); see also [33, 50, 51]. These results support recent genetic studies suggesting there is a single ERL meta-population along southeast Australia with widespread gene flow [28]. The absence of spatial genetic structure for a variety of species with a planktonic stage in this region [52, 53] indicates that patterns of connectivity are widely influenced by the prevailing circulation in this system irrespective of larval life traits [25, 33].

As a well-formed jet with few instabilities, the EAC has the potential to retain larvae within its core, transport them poleward while inhibiting across-shore transport; and hence, prevent the supply of larvae to the continental shelf-break for subsequent settlement. A modelling study used particle trajectories to show that a jet has a retaining effect and can only leak particles when it experiences instabilities [54]. The EAC can act as a jet, and its potential to inhibit exchange between offshore and coastal waters has been inferred from particle trajectories that rarely move across the EAC [33]. Supporting the hypotheses of 1) a jet inhibiting across-jet transport, and 2) the EAC acting as a jet, we find that the observed low settlement in the north coincides with a latitudinal range (28–31.9°S) where the EAC is more often a continuous poleward stream. Conversely, in the southern section, encompassing the region where the EAC typically separates from the continent, there is high settlement (Fig 2).

From the observed and simulated patterns of low and high settlement up and downstream of the EAC separation respectively; we infer that settlement of ERL in the region where the EAC separates (most frequent at 32 and 33°S, based on 21 years of OFAM BRAN 3p5 data, Fig 2C) benefits from two processes: 1) the advection of larvae by the EAC from upstream spawning locations, and 2) the occurrence of flow meandering that facilitates transport into the continental shelf-break. Similarly, along the coast of Western Australia, the poleward flowing Leeuwin current increases the downstream transport of larvae [55], and settlement in southern regions when the current is strong [56].

Although low, the occurrence of settlement in the north of the region indicates that the EAC is not always coherent within this section of the coast but it also meanders and is interrupted by the presence of eddies. In fact, within this section (28–32°S), the EAC sometimes deviates from an alongshore pathway, having its core located more than 70 km away from the coastline 20–40% of the time over the 21 year period (Fig 2). The EAC meandering and formation of eddies as far north as 25°S has the potential to induce transport from offshore into the continental shelf-break, and flow reversals which would in turn enable settlement in the northern section of the region. Monitoring locations are distributed up and downstream of the EAC separation and are therefore able to capture settlement associated with different circulation regimes.

#### Effect of mesoscale eddies on settlement

The impact of eddies retaining larvae and shaping dispersal patterns has been well documented in other regions [57–61]. For instance, in New Zealand, high abundances of rock lobster larvae observed in plankton samples coincided with an area of strong eddy circulation [62]. Similarly, the presence of eddies offshore of the Middle Florida Keys induces counter-current anomalies and influx of pueruli [63]. Moreover, [64] suggest that frontal eddies off southeastern Australia have the potential to entrain and retain fish larvae over the pelagic phase allowing recruitment back to the coast. Also, within our study region, modelling studies have shown that eddies retain particles [33], and that areas with more eddy activity relate to higher settlement [25].

This study corroborates the role of eddies in the distribution of larvae and in their role enhancing delivery to the continental shelf-break. Trajectories of larvae arriving to the continental shelf-break in pulses (i.e. in considerable quantities over a few days) consistently show a curved pathway that indicates their transport within an eddy from offshore towards the continental shelf (Fig 7). In addition, settlement peaks along the coast are associated with regions where eddies prevail (Fig 6).

### Temporal variability in settlement

Our results show that our model has useful capacity for the prediction of the inter-annual scale of settlement (Fig 4) and of dominant periodicities in settlement variability (Fig 5) over broad spatial scales (i.e., total settlement along southeast Australia). The model captures annual settlement over time periods of up to 10 years as well as year-to-year observed fluctuations in settlement for the 21-year monitoring period (1995–2015). Thus, larval dispersal simulations aid in identifying the physical processes associated with settlement. Years with low settlement (e.g. 2005) tend to have a first EOF that shows a coherent EAC, whereas years with high settlement (e.g. 1999) have first EOF’s that show a velocity field with large spatial variability and eddies (Fig 6). Thus, the dominance of a coherent EAC jet during the period when larvae settle (8–12 months after spawning) consistently translates into years of low settlement; while years when flow is eddy dominated have high settlement (Fig 6). In agreement, we find that the delivery of larvae to the continental shelf-break by eddies alone contributes ~ 30% of simulated settlement in a year.

Although circulation variability patterns relate well to temporal settlement patterns of the ERL along the southeast coast of Australia, there are some years when modelled settlement departs from the observed, suggesting variability in settlement is not determined by ocean circulation alone. We find different inter-decadal trends in observed and simulated settlement, which may be related to the changing size of the breeding stock. An increase in recorded catch per unit effort (CPUE) suggests growth of the breeding population, which could in turn induce the positive trend in observed settlement. The CPUE between 1995–2005 remains relatively constant with highest values of ~2.5 kg per trap-month; in following years CPUE increased continuously, at least until 2010, reaching ~4.5 kg per trap-month in 2007–2008 (Montgomery and Liggins 2013). Interestingly, observed and simulated annual settlements are significantly correlated when partitioned into two periods (1995–2005, and 2010–2015) that disregard the years when CPUE increased monotonically (2006–2010). Hence, the assumption of a constant spawning stock in our simulations could be provoking the dissimilarities we find between observed and simulated long-term settlement trends. Indeed, observed and simulated year-to-year settlement differences, in which long-term fluctuations are minimized, are in better agreement. Thus, incorporating spawning population size on larval dispersal simulations could yield more accurate estimates of annual settlement abundance.

In addition, our simulations consider that mortality is uniform in space and time, and do not include larval responses to the environment (e.g., behavioural or physiological responses). Little is known about changes in the interaction of larval traits with a changing oceanic environment over long time periods (inter-decadal), or the extent to which biological factors affect dispersal. [25] showed that under the A1B future climate change scenario, temperature changes will be favourable for lobster larval survival and consequent settlement particularly at 30°S and further south along southeast Australia. Conversely, for the northern region of the ERL range the seawater temperatures for larvae are increasingly less favourable [8]. Similar climate driven changes (e.g., temperature increases) have occurred in the recent decades along other Western Boundary Currents [65]. Therefore, the impact on settlement of factors that are not considered in our simulations (e.g., effect of temperature on larval survival), may cause the observed (positive) long-term trends in settlement in the south-eastern regions of this study.

### Model limitations

This study aims to model the delivery of larvae to the continental shelf-break, and shows that the arrival of pueruli to the coast is influenced by meso-scale circulation; nevertheless, local hydrodynamics, not accounted for in our simulations, may also influence the delivery of larvae and subsequent larval settlement. For instance, [66] found that fluctuations of spiny lobster settlement along the Mexican-Caribbean reflect the influence of mesoscale oceanographic processes, but the impact of waves must be considered over smaller time-scales to estimate settlement within reef lagoons. Similarly, winds that induce onshore transport have been correlated with high settlement [18, 67]. The role of other physical processes on cross shelf recruitment have also been identified in a number of recent studies correlating the settlement and recruitment of spiny lobsters with oceanographic and climatological variables including stokes drift and local wind forcing [10, 66, 68, 69].

In addition, horizontal swimming behaviour of late-stage larvae and pueruli may also play a role in determining arrival on the coast. For instance, [70] find that mid-stage larval distributions of mid-stage larvae of *J*. *edwardsii* are well described by passive drift alone, but other mechanisms inducing shoreward transport are needed to explain the shoreward movement of the subsequent late-stage larvae. Furthermore, spiny lobster pueruli are known to be capable of extended periods of cross-shelf swimming of tens to hundreds of kilometres required to reach shallow coastal reefs where they settle [35, 71, 72]. In this study we have not implemented any of these more complex scenarios and yet the predictive capability of settlement inter-annual variation across all sites is significant over decadal periods; however, it prevents the simulations from predicting variation in settlement at individual locations.

### Implications for management

Simulated larval trajectories show connectivity throughout southeast Australia (Fig 4) in agreement with genetic studies, which identify a single ERL population in the region. This implies that estimates of settlement over broad spatial scales (total across southeast Australia) are useful to set catch quotas. The relationships we find between the EAC coherence, presence of eddies, and the observed spatio-temporal patterns of settlement suggest that proxies for broad spatio-temporal patterns settlement can be developed from products such as satellite altimetry that capture the EAC variability and the meso-scale eddy field. Such proxies have potential to provide fisheries managers with a useful tool to anticipate broad settlement patterns, and future recruitment into the fishery. This would allow greater capability to set fisheries quotas and sustainably manage this highly valued fishery into the future.
