# Correction: Mesoscale circulation determines broad spatio-temporal settlement patterns of lobster

**DOI:** 10.1371/journal.pone.0214996

**Published:** 2019-04-02

**Authors:** Paulina Cetina-Heredia, Moninya Roughan, Geoffrey Liggins, Melinda A. Coleman, Andrew Jeffs

The images for Figs 4, 5, 6 and 7 are incorrectly switched. The image that appears as Fig 4 should be Fig 7; the image that appears as Fig 5 should be Fig 4; the image that appears as Fig 6 should be Fig 5; and the image that appears as Fig 7 should be Fig 6. The figure captions appear in the correct order.

**Fig 4:**
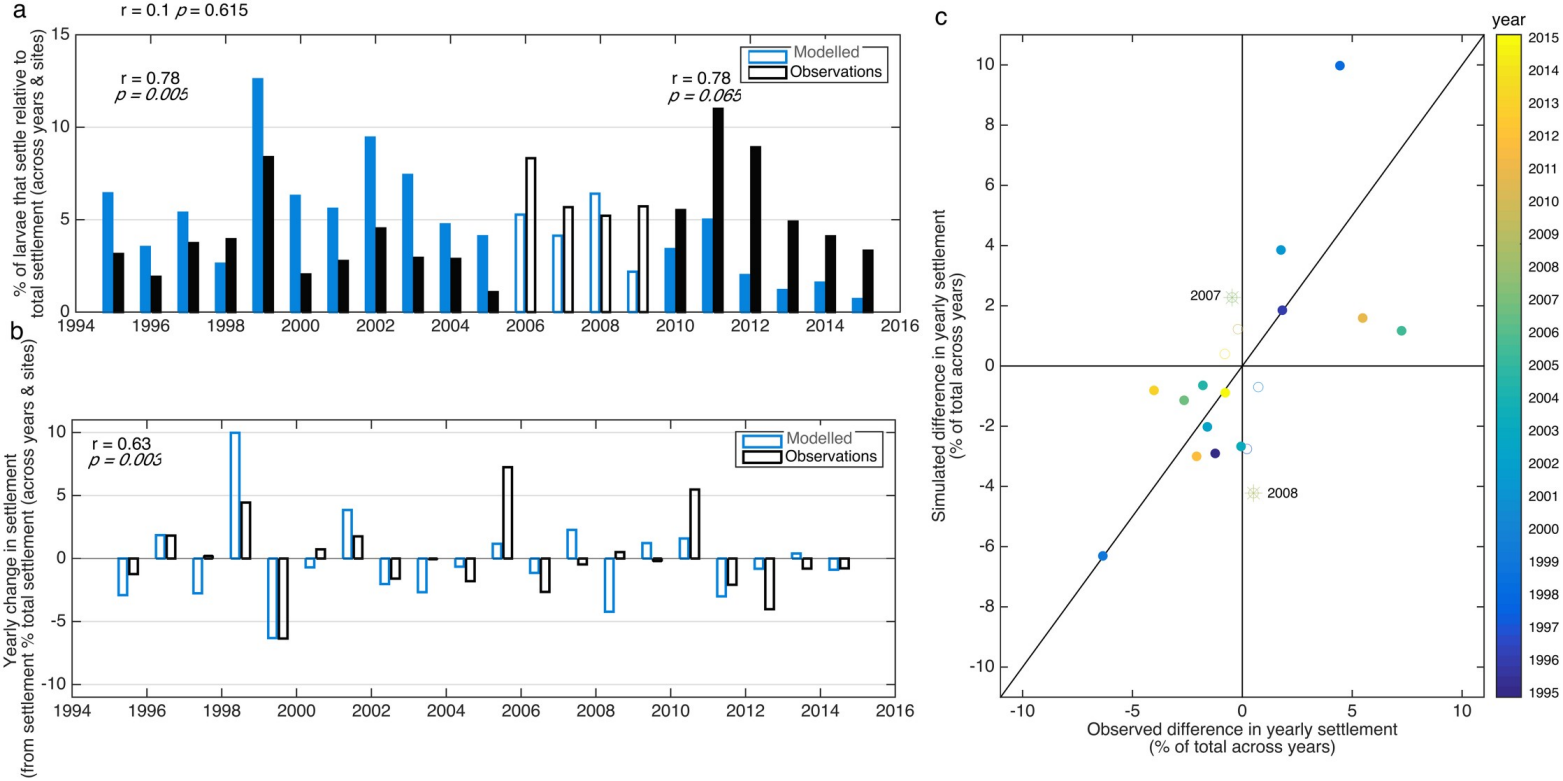
a) Twenty-one year (1995–2015) time series of observed (black) and simulated (blue) settlement added across monitoring locations, correlation coefficients (r) and corresponding *p* values are shown for correlations between 1995–2015 (r = 0.1, *p>>0*.*05*), 1995–2005 (r = 0.78, *p < 0*.*01* filled bars), and 2010–2015 (r = 0.78, *p = 0*.*065* filled bars). Open bars are used for 2006–2009 to facilitate distinction of decadal periods before and after, for which correlation coefficients and *p* values are included. b) Time series of observed and simulated yearly change in settlement, correlation coefficient (r = 0.63, *p < 0*.*01*). c) Observed vs. simulated yearly differences in settlement; filled symbols indicate years when observed and simulated differences agree (i.e., either both increase or decrease), while empty symbols indicate when they show opposite trends (i.e. one decrease and the other increases and vice versa); years with largest discrepancy are emphasized with an asterisk. Settlement and differences in settlement are presented in percentage relative to total settlement across all years for the observed and simulated settlement respectively.

**Fig 5:**
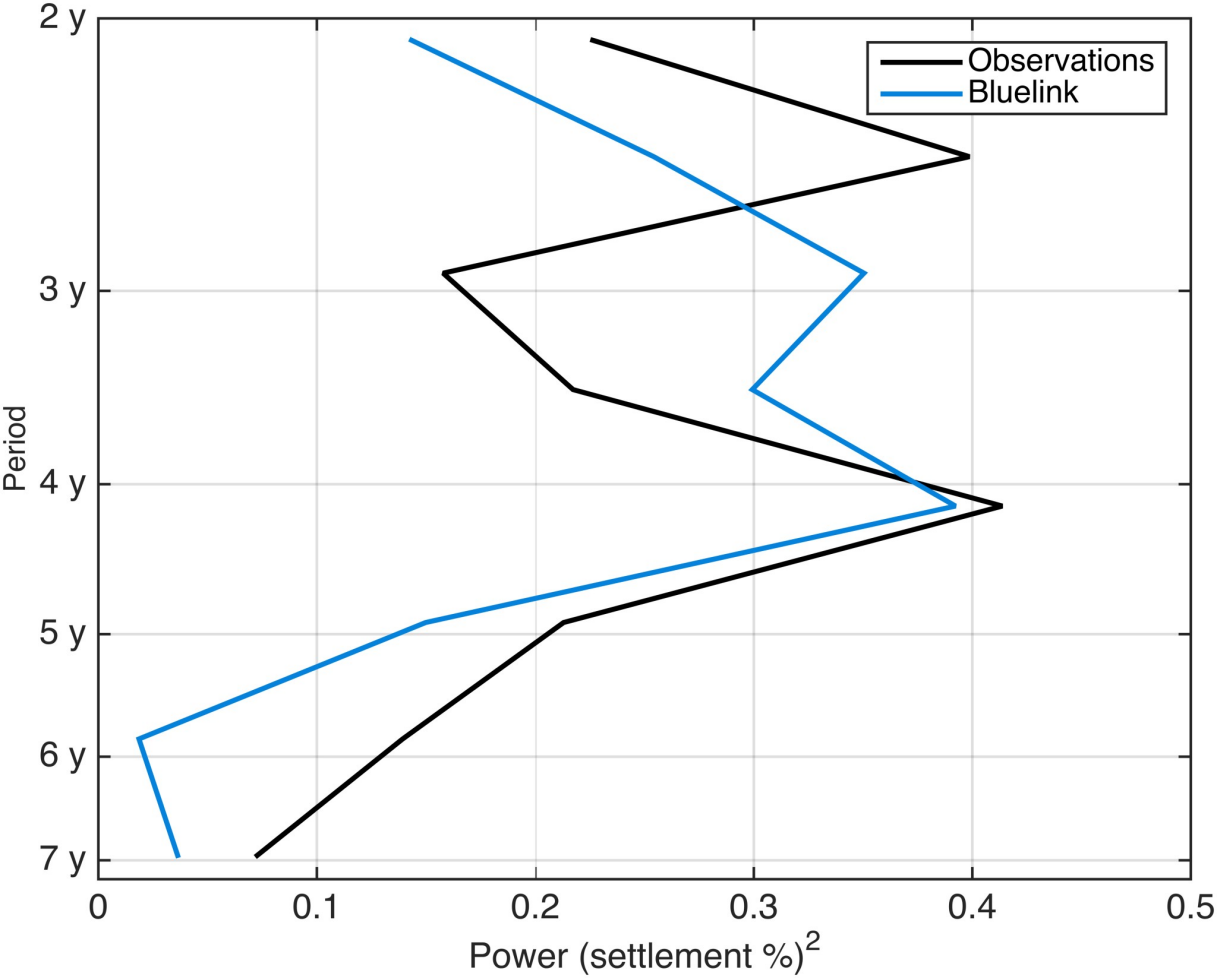
Global wavelet spectrum of simulated and observed settlement showing peaks of energy between 2–3 years and at 4 years.

**Fig 6:**
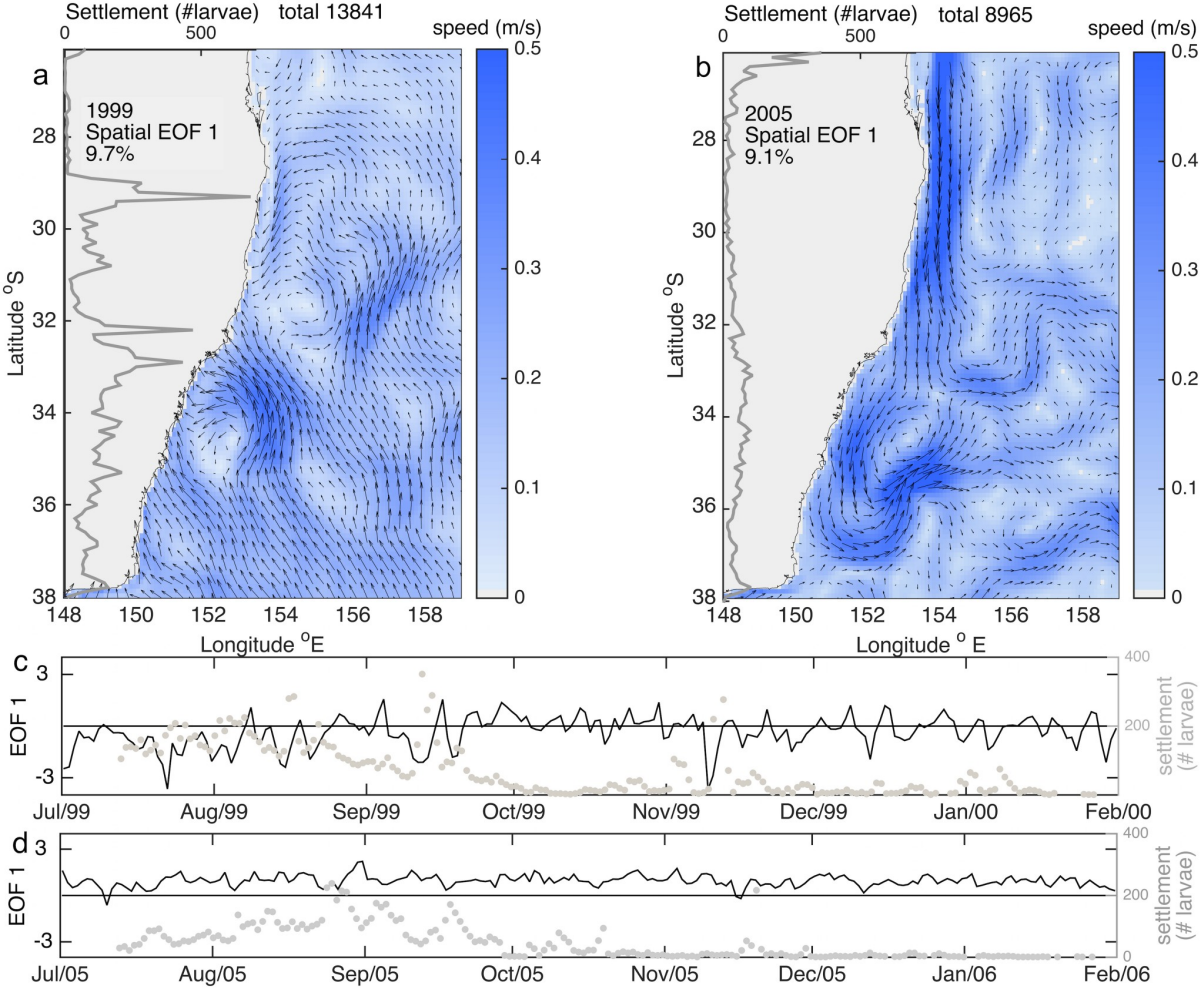
a-b) Spatial structure of the first mode of the Empirical Orthogonal Functions (EOFs) showing speed (colorbar) and velocity fields (arrows) for settlement time period in 1999 (a) and 2005 (b). Superimposed is settlement at each 0.1 latitude degree (grey line). c-d) temporal structure of the first mode of the Empirical Orthogonal Functions (EOFs) for the settlement time period in 1999 (c) and 2005 (d); the left hand axis shows settlement over time across all latitudes (grey dots).

**Fig 7:**
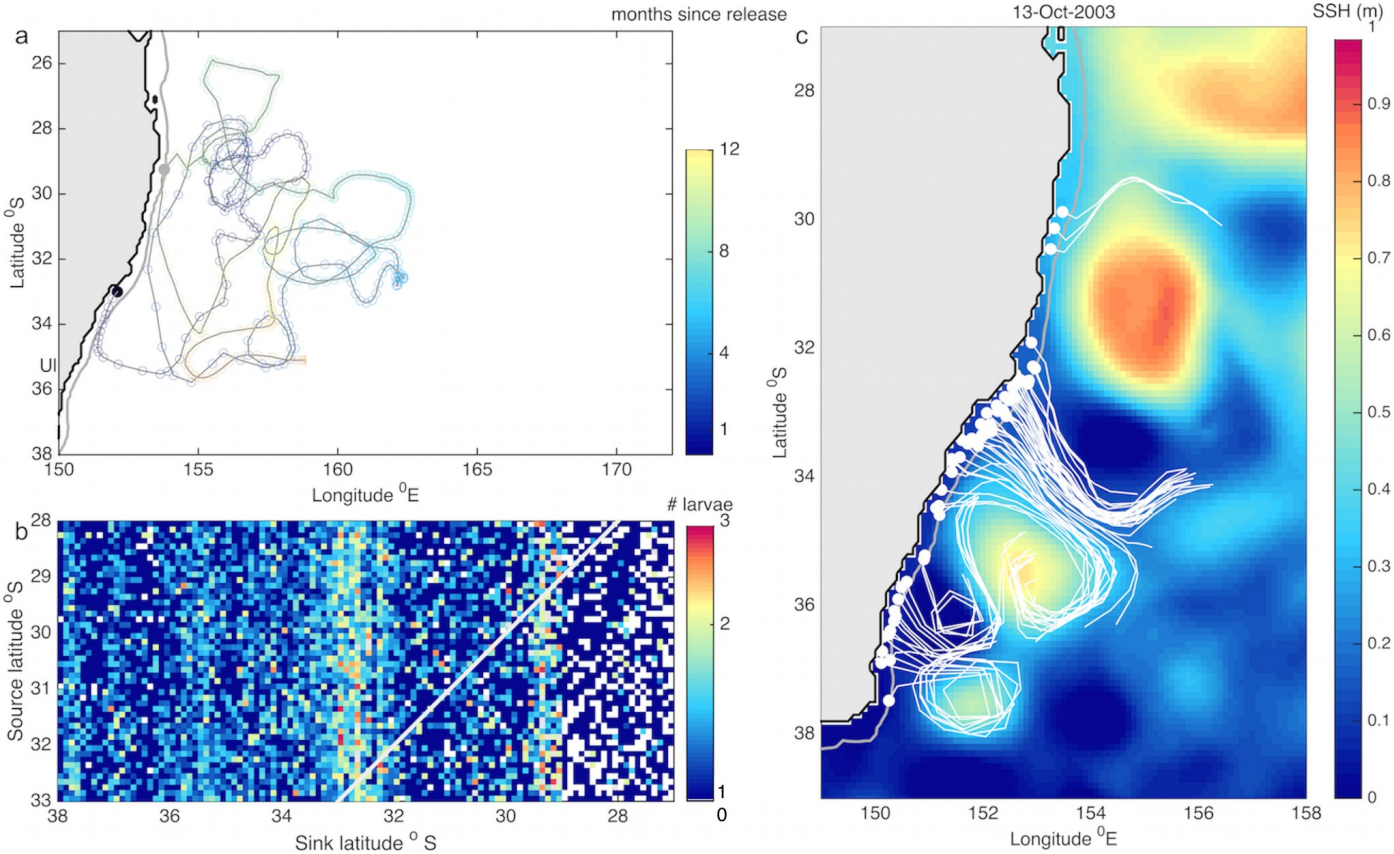
a) Trajectory of a particle that settles upstream, the black and grey dots show the release and settlement locations respectively, the particle trajectory is shown for 12 months rather than being truncated at settlement, i.e., at ~9 months after release). b) Mean connectivity matrix (1995–2015), the white indicates values that correspond to self-recruitment (the source and sink latitudes are the same), the colorbar indicates the number of larvae in logarithmic scale. c) Particle re-circulation within eddies and transport into the continental shelf break. Snapshot on the 13/October/2003 showing a simulated settlement pulse and preceding particle re-circulation within eddies with Sea Surface Height (SSH) on the background, dots indicate the position of particles at settlement and lines show their trajectories in the previous 10 days; only particles that reach settlement are shown.

## References

[1] Cetina-Heredia P, Roughan M, Liggins G, Coleman MA, Jeffs A (2019) Mesoscale circulation determines broad spatio-temporal settlement patterns of lobster. PLoS ONE 14(2): e0211722 10.1371/journal.pone.0211722 30707747 PMC6358102

